# The complete chloroplast genome of Plateau herb *Chesneya acaulis* (Fabaceae)

**DOI:** 10.1080/23802359.2021.1878955

**Published:** 2021-02-17

**Authors:** Li-Yun Nie, Ai-Hua Wang, Lei Duan, Hong-Feng Chen, Fa-Guo Wang

**Affiliations:** a Key Laboratory of Plant Resources Conservation and Sustainable Utilization, South China Botanical Garden, Chinese Academy of Sciences, Guangzhou, China; b College of Life Sciences, University of Chinese Academy of Sciences, Beijing, China; c Key Laboratory of Environment Change and Resources Use in Beibu Gulf, Ministry of Education, Nanning Normal University, Nanning, China

**Keywords:** Chloroplast genome, Fabaceae, *Chesneya acaulis*, Plateau herb

## Abstract

*Chesneya acaulis* is a perennial herb, which restricts in Xizang (Tibet) of China, Afghanistan, and Pakistan. The complete chloroplast genome was sequenced using the Illumina Hiseq X-Ten platform. The genome lacks an inverted repeat (IR) region, containing 75 protein-coding genes, 29 tRNAs genes, and 4 rRNAs. The overall GC content is 34.6%. A phylogenetic tree based on the whole chloroplast genomes of 15 species indicated that *C. acaulis* had a close relationship with the genus *Hedysarum*, and it nested in the inverted repeat-lacking clade (IRLC), of the subfamily Papilionoideae (Leguminosae).

*Chesneya acaulis* (Baker) Popov, is a plateau cushion-like herb, inhabiting gravelly areas, which restricts in Xizang (Tibet) of China, Afghanistan, and Pakistan (Li 1981, 1998; Zhu and Larsen 2010). While in China, *Chesneya acaulis* only distributed in southern Xizang. Few study focused on chloroplast genome of *Chesneya*, a good knowledge in genomic information of *Chesneya acaulis* would contribute to the study of population genetics, diversity, and geological history of this genus and other plateau plants.

The fresh leaves of *Chesneya acaulis* were collected in Zhada County, Xizang (Tibet), China, and the voucher specimen were deposited in the herbaria of Northwest A&F University (WUK, collection #: Z.Y.Chang 2013166). Total genomic DNA were extracted from leaf tissue samples with CTAB approach (Doyle 1987), the genomic libraries were prepared and sequenced using the Illumina Hiseq X-Ten platform (Illumina Inc., San Diego, CA) with 150 bp paired-end reads. The resultant sequences were filtered following Yao et al. (2016), the adaptor-free reads were then assembled with SPAdes 3.11 (Bankevich et al. 2012). The complete chloroplast (cp) genome was annotated using the Dual Organellar GenoMe Annotator (DOGMA) (Wyman et al. 2004) and deposited the genome in GenBank (accession number: MW053403).

About 1.70 Gb raw reads of *Chesneya acaulis* were obtained, with coverage of 1441× and 128,629 bp in length. The cp genome lacked an inverted repeat (IR) region. The genome contained 75 protein-coding genes (CDS), 29 transfer RNA genes (tRNA), 4 ribosomal RNA genes (rRNA), within which 16 genes (*atpF*, *ndhA*, *ndhB*, *trnI-AUC*, *trnA-UGC*, *petB*, *petD*, *rpl16*, *rpl2*, *trnG-UCC*, *rpoC1*, *rps12*, *rps12*, *trnL-CAA*, *trnV-UAC*, *trnK-UUU*) had one intron, one gene (*ycf3*) had two introns. Overall GC content of the whole genome was 34.6%.

To infer the phylogenetic relationship between the newly sequenced *C. acaulis* and its related taxa, 14 chloroplast genomes were downloaded from GenBank and were applied to construct the systematic tree. We aligned these 15 cp genomes using MAFFT version 7 (Katoh and Standley 2013) and generated a maximum-likelihood (ML) tree through the program IQ-TREE version 1.4.2 (Nguyen et al. 2015). The result (Figure 1) showed that genus *Chesneya* had a close relationship with *Hedysarum*, and nested in the IR-lacking clade (IRLC), which in turn belonged to the clade of Hologalegina of papilionoid legumes as suggested by a previous study (Wojciechowski et al. 2004).

**Figure 1.**
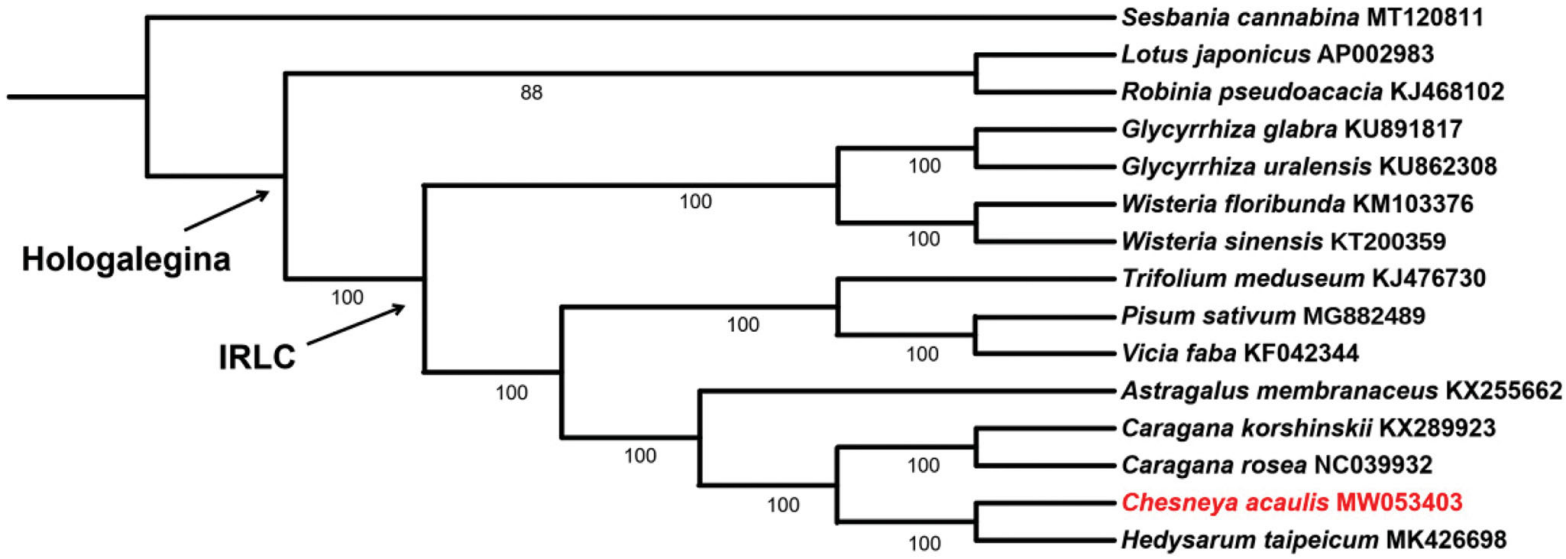
Maximum likelihood (ML) phylogenetic tree based on 15 chloroplast genomes of Fabaceae, where *Chesneya acaulis* is highlighted in red. The bootstrap values are shown below branches.

## Data Availability

The genome sequence data that support the findings of this study are openly available in GenBank of NCBI at (https://www.ncbi.nlm.nih.gov/) under the accession no. MW053403.
